# Family Caregivers’ Experiences with Tele-Rehabilitation for Older Adults with Hip Fracture

**DOI:** 10.3390/jcm10245850

**Published:** 2021-12-13

**Authors:** Patrocinio Ariza-Vega, Rafael Prieto-Moreno, Herminia Castillo-Pérez, Virginia Martínez-Ruiz, Dulce Romero-Ayuso, Maureen C. Ashe

**Affiliations:** 1 Department of Physiotherapy, University of Granada, 18016 Granada, Spain; pariza@ugr.es (P.A.-V.); dulceromero@ugr.es (D.R.-A.); 2 Physical Medicine and Rehabilitation Service, Biohealth Research Institute, Virgen de las Nieves University Hospital, 18012 Granada, Spain; 3 PA-HELP “Physical Activity for HEaLth Promotion” Research Group, Department of Physical and Sport Education, Faculty of Sports Sciences, University of Granada, 18071 Granada, Spain; 4 Ciudad de Berja Nursing Home, Berja, 04760 Almería, Spain; hermicp11@gmail.com; 5 Department of Preventive Medicine and Public Health, School of Medicine, University of Granada, 18016 Granada, Spain; virmruiz@ugr.es; 6 Center for Biomedical Research in Network of Epidemiology and Public Health (CIBERESP), 28029, Madrid, Spain; 7 Instituto de Investigación Biosanitaria de Granada (ibs.GRANADA), 18014 Granada, Spain; 8 Centre for Hip Health and Mobility, Department of Family Practice, University of British Columbia, Vancouver, BC V5Z 1M9, Canada; maureen.ashe@ubc.ca

**Keywords:** tele-rehabilitation, hip fracture, older people, family caregiver, information and communication technology

## Abstract

Background: There is a knowledge gap for implementing tele-rehabilitation (telerehab) after hip fracture. We recently conducted a clinical trial (ClinicalTrials.gov Identifier: NCT02968589) to test a novel online family caregiver-supported rehabilitation program for older adults with hip fracture, called @ctivehip. In this qualitative substudy, our objective was to use semi-structured interviews to explore family caregivers experience with the telerehab program. Methods: Twenty-one family caregivers were interviewed between three and six months after the older adults completed @ctivehip. One occupational therapist with research and clinical experience, but not involved in the main trial, conducted and transcribed the interviews. We conducted a multi-step content analysis, and two authors completed one coding cycle and two recoding cycles. Results: Family caregivers who enrolled in @ctivehip were satisfied with the program, stated it was manageable to use, and perceived benefits for older adults’ functional recovery after hip fracture. They also suggested improvements for the program content, such as more variety with exercises, and increased monitoring by health professionals. Conclusions: This work extends existing literature and generates research hypotheses for future studies to test telerehab content and program implementation.

## 1. Introduction

Loss of functional independence [1], decreased social participation [1], and reduced quality of life [2] are some of the main consequences of hip fractures. Early hospital rehabilitation with follow-up post-discharge can support older adults’ recovery of function [3]. Family caregivers play an essential role in helping older patients to complete activities of daily living (ADL) in the home setting [4,5]. The sudden and unexpected nature of hip fractures can impact both older adults and family caregivers, who as a result can experience increased stress and burden [6]. As a result, hip fracture is associated with worse overall health status in family caregivers [7]. These factors indicate a need for new post-discharge management strategies [8,9] to improve older adults ‘ function post-hip fracture and reduce caregiver stress [10].

There are barriers to delivering rehabilitation after hip fracture, such as limited access to health professionals after discharge to home. Telerehabilitation (telerehab) is a promising management strategy to support recovery after discharge, and may be especially important in rural and remote areas with limited access to in-person rehabilitation [11]. Of note, there has been an increase in remote delivery of health care because of the SARS-CoV-2 (COVID-19) pandemic [12]. Based on previous studies, telerehab for musculoskeletal injuries or conditions were effective for improving physical function, quality of life, and psychological factors [13,14,15,16]. However, there is limited evidence for: (i) the effect of telerehab for older adults after hip fracture [17], and (ii) the inclusion of family caregivers in telerehab for hip fracture [18]. We focus on family caregivers to support older people using information and communication technologies (ICT), and to provide support for the telerehab program. Involving family caregivers in telerehab also addresses their request for more information on the recovery process [19]. Thus, we aimed to address these knowledge gaps [17] by designing and testing a telerehab program called @ctivehip for older adults with hip fracture and their family caregivers.

We previously published results from the main trial [20]—a choice-based multiple methods clinical trial comparing @ctivehip telerehab with home-based in-person rehabilitation for functional recovery of older adults with hip fracture [21]. The @ctivehip intervention consisted of: (i) web-based information to increase family caregivers’ knowledge and skill development; (ii) a supported exercise and ADL program for older adults (delivered by the family caregiver); (iii) a specific section on family caregivers’ health; and (iv) an option for family caregivers to video conference with health professionals.

The aim of the present exploratory study was to describe family caregivers experience with the @ctivehip telerehab program. We anticipated that family caregivers’ feedback could be used to refine the intervention by identifying implementation opportunities and challenges from a person-centered approach.

## 2. Materials and Methods

### 2.1. Design

This was a substudy of a multiple methods clinical trial (Clinical Registration NCT02968589). We previously published the results of the main study (quantitative findings, primary outcome functional level) [20], and patients’ and family caregivers’ overall experience with hip fractures (qualitative findings) [22]. In this second qualitative study, we aimed to synthesize experiences of the family caregivers of older adults with hip fracture enrolled in the @ctivehip telerehab program. We were guided by the principles of interpretive description [23] when designing the interview guide, conducting interviews, and synthesizing findings.

### 2.2. Participants

**Figure 1:**
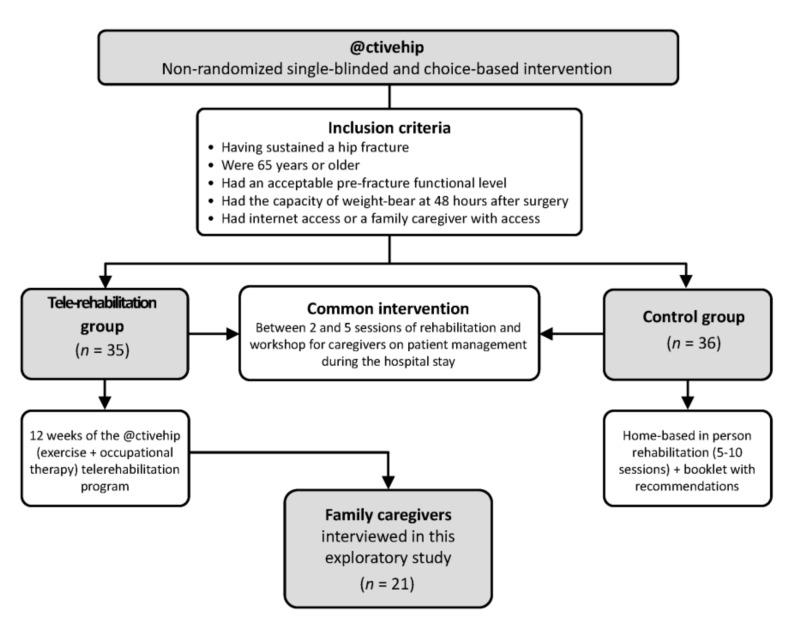
Flowchart for caregivers recruitment.

A description of participant recruitment is provided elsewhere [21]; please see Figure 1. At the final assessment of the main trial (conducted at three months after hip fracture surgery), family caregivers were invited to participate in semi-structured interviews. For the present study, we summarize responses only from participants who requested @ctivehip (e.g., the intervention group). There were 23 family caregivers who agreed to participate and signed the study consent form. When we telephoned family caregivers three months later, two family caregivers did not answer the telephone after several attempts. Thus, 21 family caregivers representing 21 older adults with hip fracture were interviewed for the present study.

### 2.3. Semi-Structured Interviews

Between October 2017 and December 2018 an occupational therapist (OT), with related clinical experience but not involved in the main clinical trial, conducted the telephone interviews between three and six months after participants finished the main clinical trial. The virtual interviews were scheduled when family caregivers were at home, to minimalize potential distractions. The OT recorded and transcribed the interviews within two days after each interview and kept field notes for reference during the analysis process. The interview guide is provided in Appendix A. During the interviews, the OT encouraged discussion using prompts such as “please explain how you did it”, “tell me more about it”, and follow-up questions to encourage participants to provide more details of their experience. To explore family caregivers’ perceptions about the utility of, and satisfaction with, the telerehab program we asked participants to rate their experience using a scale of 0–10 points (0 = not useful and 10 = very useful; 0 = lowest level of satisfaction and 10 = highest level of satisfaction). On average, interviews lasted 18 (range 1–22) min.

### 2.4. Data Analysis

We followed the recommendations of Graneheim and Lundman [24] to conduct a multi-step content analysis. Two authors P.A.-V. and R.P.-M. first read the transcripts (in Spanish) several times. Following this they met three times to review data, create a coding framework, and synthesize findings. The process involved one coding cycle and two re-coding cycles [25] to increase confidence in the response classification. During this process, the authors identified meaning units, sorted them into subcategories, and then categories. Data (categories and quotes) were translated into English by the first and third authors (native Spanish speakers with proficiency in English) and reviewed by the last author (native English speaker). There was lengthy discussion between authors to ensure the cultural context was considered.

### 2.5. Trustworthiness of the Findings

We included several processes to increase trustworthiness of study findings [26]. First, the OT who conducted the interviews was experienced in the management of older adults with hip fracture, however they were not involved in the main clinical trial. Second, the interviews were recorded (with permission) and the interviewer took field notes. They also checked in with participants during the interview to clarify responses. Third, data were transcribed by the OT and one assistant within two days of the interview. Fourth, an audit trail was maintained throughout the process to summarize analysis steps and decisions. Fifth, a representative subgroup of participants checked a summary from emerging themes and quotes, and they were invited to add or change information. Finally, investigator triangulation was applied [27]. The first author (dual-trained physical therapist (PT) and OT with a doctoral degree) and third author (experienced OT with a research MSc) analyzed the data. Following this, the last author (PT and professor with a doctoral degree) reviewed the findings and discussed them multiple times with the first author.

We used NVivo 10 (QSR International, Doncaster, Australia) during data analysis to assist with data management (i.e., managing files, coding process, and analysis). We present participants age and scores for telerehab program utility and satisfaction using median (q25–q75) values.

## 3. Results

### 3.1. Demographics Characteristics

Twenty one family caregivers (16 women and five men; median (q25–q75) age 50 (43–54) years) participated in the present study. Most family caregivers were the offspring of the patients with a hip fracture (18/21; 86%) supported by other family caregivers (15/21; 71%), who lived with the patient (14/21; 67%), and more than half were also working (14/21; 67%) part-time or full-time. The median age of older adults with hip fracture was 78 (73–82) years; most were women (76%), and their functional level at the end of the telerehab program (12 weeks) was similar to their pre-fracture functional level assessed through the Functional Independence Measure (FIM) [28]. A detailed description of family caregivers’ characteristics is provided in Table 1.

### 3.2. Adherence to the Telerehab Program

Ten of twenty-one caregivers completed the program as intended (high fidelity at 12 weeks), and an additional six participants completed 8 weeks or more of the program (76% in total). Half of the caregivers (10/21; 48%) stated their older family member completed the program, and then continued doing the exercises for a few more months. However, the remaining caregivers reported their family member stopped doing the exercises before the end of the 12 weeks. Most family caregivers (20/21; 95%) expressed 12 weeks was long enough to learn the program, or they believed their family member did not require rehabilitation beyond 12 weeks.

*“…We spent about one month doing the exercises with her. Afterwards she continued doing the exercises alone, but I think she did not do them for twelve weeks… She stopped when she felt she did not need to do [any] more…”*

(Caregiver 8)

The caregivers with lower adherence to the telerehab program (5/21) were all women, older than 50 years of age (range; 52–56 years) and had no support from other caregivers (4/5; 80%) or the support of one additional caregiver to help the patient with ADLs (1; 20%). From the five caregivers with lower program adherence, two were unemployed, two worked part-time, and one worked full-time.

### 3.3. Categories

**Figure 2:**
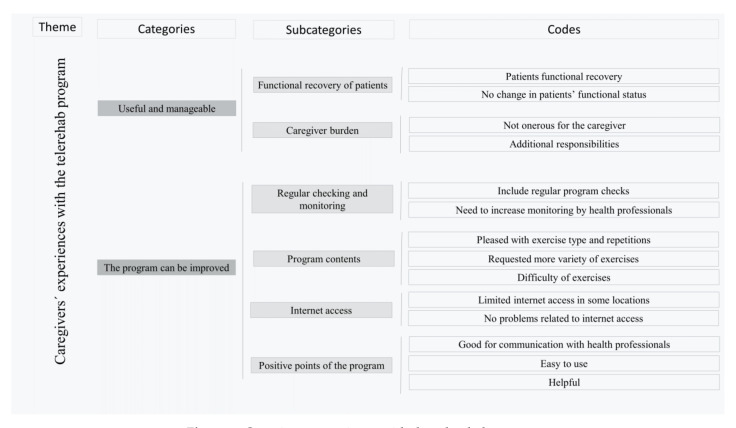
Caregivers experience with the telerehab program.

There were two categories generated during data analysis: (1) the telerehab program was perceived to be useful for older adults’ functional recovery without being onerous for family caregivers; and (2) there was room for improvement in the telerehab program. A visual summary of the main findings is provided in Figure 2.

#### 3.3.1. The telerehab clinical program: Useful and manageable

Caregiver support was essential for implementing @ctivehip. Overall, family caregivers were highly satisfied with the telerehab program and rated it useful (median (q25, q75): 8 (8-9)/10 points for utility and 9 (8–9.5)/10 points for satisfaction. Though the use of the program required more time and additional responsibilities for the caregivers, most participants stated there was no additional caregiving burden with using @ctivehip:

*“…At first, it took a while but then we [created a] habit. I came home from work and connected the tablet. I helped her with the exercises that seemed more difficult.”*

(Caregiver 12)

*“…You need to [make time] to use the program… my brother and I organized [it] between us and it was not too much burden…”*

(Caregiver 15)

Although most caregivers noted improvement in patients’ health after using @ctivehip, a few family caregivers reported no change in their family member’s function with the program:

*“…She could almost not walk and now she is completely independent…”*

(Caregiver 15)

*“…We started in the hospital, then he spent 3 months in my house doing the exercises and then he went home and continued doing them but without [the] internet… He already memorized the exercises and keeps doing them because he says they are good for him and that the doctor told him he could continue doing them every day… I would say that he is better than before the surgery…”*

(Caregiver 4)

*“…No, she won’t be the same person anymore… She is having a setback in general…”*

(Caregiver 3)

#### 3.3.2. Room for improvement

Based on the caregivers experience, there was an identified need to develop new approaches for health professionals to support them during the recovery process. Study participants provided valuable insights for how best to implement @ctivehip in the future. Although family caregivers were aware they could request a video-conference session with a health professional, they preferred a regular check-in within the telerehab program to verify everything was proceeding as planned. Some family caregivers further suggested regular health professional monitoring to support older adults’ confidence with completing exercises:

*“…I [missed] that the staff would call us from time to time to ask us how we were doing and to test if we were doing the exercises properly. …I know we could have called to ask for a video conference, but we felt in some way alone…”*

(Caregiver 13)

There were some differences among suggestions to improve the program content. Many family caregivers suggested the program should include more variety in the exercises to reduce the risk of boredom, and possibly increase older adults motivation. In contrast, some family caregivers were pleased with the repetition of the exercises, as it made them easier to remember:

*“…All the exercises were very similar and became very monotonous and repetitive after the first week…”*

(Caregiver 18)

*“…I’m not an expert but I believe the exercises were [good] because my father memorized them every week and if he did not have internet one day, he would do it himself…”*

(Caregiver 16)

Family caregivers rated the program’s level of difficulty as either low or average. Nevertheless, eight family caregivers stated their family member avoided using weights to perform some exercises due to low confidence:

*“…She did not do some exercises because she did not feel safe. For example, the exercises with the weights… She did the exercises but without weights…”*

(Caregiver 13)

Almost half of the caregivers stated they liked most components of the telerehab program, while seven family caregivers liked everything about the program. Constructive feedback on the program included difficulty accessing internet in some locations, and the exercises were repetitive (and possibly created boredom). Family caregivers also reported positive attributes of the program, such as usefulness for functional recovery (helpful and well-presented information, and ease of use).

## 4. Discussion

Telerehab is a rapidly growing mode of health care delivery, but there are known gaps for using ICTs for older adults with hip fracture [17]. Recognizing ICT barriers in the early discharge recovery period, we engaged family caregivers to facilitate the adoption of remote delivery of health care after hip fracture. Here we provide a detailed description of family caregivers experience using a telerehab program called @ctivehip. Overall, family caregivers reported a high level of satisfaction with the program, stated it was manageable to use, and reported it was useful for functional recovery. Importantly family caregivers provided valuable insights for future program content and implementation to strengthen delivery and uptake of the intervention.

Although participants chose to receive the telerehab program, our sample was similar to other studies where the caregiving was mainly provided by women [4,5] at middle-age [29,30] who were adult children, [6] and were supported by other family caregivers. [4,30] In our study most family caregivers worked full-time or part-time, in contrast to other studies where caregivers were mostly unemployed. [5,6] These characteristics can be influenced by social and cultural norms and by the organization of the social and healthcare systems. Our work provides a novel perspective on caregiving while employed showing similar adherence to the program between caregivers who were unemployed, and those with part-or full-time work. Further, caregivers with lower adherence to the program reported lower support from other caregivers. This finding generates hypotheses on support (amount and type) needed by caregivers to deliver and manage the program. Although telerehab in general may be cost-effective [31], future research is needed to determine the acceptability, costs, feasibility, and (cost) effectiveness of telerehab considering the caregivers perspective in a program like @ctivehip.

We observed a high level of satisfaction and perceived usefulness for family caregivers with the @ctivehip program. These factors may contribute to users’ motivation to adopt and persist with the program, a key component of the technology acceptance model [32]. Our findings are similar to other ICT studies based on health communication and family caregivers’ health literacy and caregiving skills [33,34]. Of note, in our study family caregivers did not perceive the program as onerous, even with the additional time commitment. Further, in our other qualitative study from @ctivehip, these same family caregivers in the intervention group reported lower levels of stress and anxiety and requested less social and health services compared with caregivers of patients who received only a few sessions of in-person rehabilitation [22]. An explanation for these findings may be related to the perceived benefits of the telerehab program: it was an opportunity for family members to receive education and skills training to prepare for the recovery process. Other studies reported family caregivers wanted role clarification and active participation in their family members recovery [10,18,22]. It is possible caregivers’ observation of older adults’ functional improvement with the program may have benefited both the caregiver (improved self-efficacy in caregiving) and the older adult with hip fracture (adoption and use of the program, mastery with exercises, improved self-efficacy, etc.) However, as we did not measure the effect of these factors in our study, we can only generate hypotheses on the “active ingredients” [35] or behavior change techniques associated with the telerehab program or its delivery.

Family caregivers requested regular communication with health professionals after hospital discharge (using a person-centered approach) to update and progress rehabilitation plans. At present we do not know if the family caregiver “regular check-ins” need to be face-to-face or via ICTs. Technology may provide opportunities for more frequent communication with family caregivers and patients, especially during the transition back home [34]. An automatic system with personalized patient/caregiver feedback and monitoring to enhance motivation could be considered, similar to a system described in a pilot study of older adults with hip fracture [36]. We also recognize some patients and caregivers prefer face-to-face interactions [37]. Thus, health education should be individualized to each person, and consider internet resources available, motivation (habitual processes, emotional responses, and analytical decision-making), capability (knowledge and skills) and opportunities (context) of the patients, caregivers, and health professionals [37,38].

Family caregivers in our study requested more and varied exercise options within @ctivehip but, as some of the caregivers recognized, some patients did not use weights during the sessions for safety reasons. More frequent communication with health professionals could have addressed concerns, develop strategies, and explain why using weights (if possible) was recommended to increase strength and physical condition. It is difficult to know how the content of our telerehab program [21] compares with other published studies in this area [36,39,40] as previous work only provided a brief description of interventions.

We acknowledge that only half of participants completed the full program but overall, three-quarters completed eight weeks or more of the intervention. Reasons to explain the lack of fidelity to the program could be boredom related to the repetitive nature of the core exercises, participants higher level of function (pre-and post-intervention) reported in the main trial [20], or older adults (and family members) may have stopped the program when they felt independent in completing ADLs. The unified theory of acceptance and use of technology (UTAUT) describes factors which impact on technology adoption and use, such as social influence, effort expectancy, and performance expectancy [41]. Considering the UTAUT, possible elements to increase @ctivehip program adherence include reimagining work organization, with the inclusion of periodic health monitoring [42]; clear communication with patients and caregivers and a detailed explanation of the program [18,29]; discussing caregivers expectations [43]; and/or specific information on program progression (if/when appropriate). For example, progressing exercises with weights, if possible. Although we collected some implementation metrics, our future work needs to discern the optimal “dose” of the program, individualized for older adults following hip fracture.

### Strengths and Limitations

We note some limitations within our study. First, due to the study’s inclusion criteria, the older adults with hip fracture did not have cognitive impairment. Future studies should consider expanding the inclusion criteria to reflect a wider population of people who fracture their hip. Second, we conducted interviews three to six months after the telerehab program ended, and this delayed timing may have impacted family caregivers’ responses. Third, we conducted short interviews although provided prompts and cues to explore caregivers’ experience using the program. Fourth, group assignment was by choice, therefore participants who wanted to receive the telehealth program may have a different experience. Although many caregivers liked the program there were some perceived limitations such as the limited variety of exercises and monitoring. Fifth, we did not capture psychosocial factors such as caregivers’ self-efficacy with delivering the telerehab program, but we recognize behavioral factors (for the older adult and family caregiver) are important to include in future research. Despite these limitations, our study provides valuable information to extend the limited evidence for ICTs for older community-dwelling adults with hip fracture [44,45] and their family caregivers [33].

## 5. Conclusions

To our knowledge, this is the first study to provide a detailed description of family caregivers’ experience with a post-hip fracture telerehab program delivered before the SARS-CoV-2 (COVID-19) pandemic. Family caregivers who enrolled in the @ctivehip telerehab program were satisfied with the program, stated it was manageable, and reported perceived benefits for older adults’ functional recovery after hip fractures. Family caregivers also provided helpful feedback to enhance program content and its delivery. Taken together, this work extends existing literature, and generates research hypotheses for future studies to test telerehab content and program implementation.

## Figures and Tables

**Table 1:** Caregivers’ characteristics.

Age	Gender	Relationship to Patient	Living with the Patient	Employment	Support of Other Caregivers (Number)
44	Woman	Daughter	No	Part-time	Yes (2)
53	Woman	Wife	Yes	Unemployed	Yes (1)
64	Woman	Daughter	Yes	Unemployed	Yes (2)
56	Woman	Daughter	Yes	Part-time	No (0)
42	Woman	Daughter	Yes	Part-time	Yes (3)
41	Woman	Daughter	Yes	Full-time	Yes (1)
38	Man	Son	No	Full-time	No (0)
40	Woman	Daughter	No	Full-time	Yes (1)
50	Woman	Daughter	Yes	Unemployed	No (0)
45	Woman	Daughter	Yes	Unemployed	Yes (2)
54	Woman	Niece	Yes	Full-time	No (0)
55	Woman	Niece	No	Part-time	No (0)
53	Woman	Daughter	No	Unemployed	Yes (2)
50	Man	Son	No	Unemployed	Yes (2)
54	Woman	Daughter	Yes	Part-time	Yes (1)
52	Woman	Daughter in law	Yes	Unemployed	No (0)
43	Man	Soon	Yes	Full-time	Yes (1)
44	Woman	Daughter	Yes	Full-time	Yes (1)
53	Man	Son	Yes	Part-time	Yes (2)
51	Man	Son	No	Full-time	Yes (3)
40	Woman	Daughter	Yes	Full-time	Yes (2)

**Figure A1:**
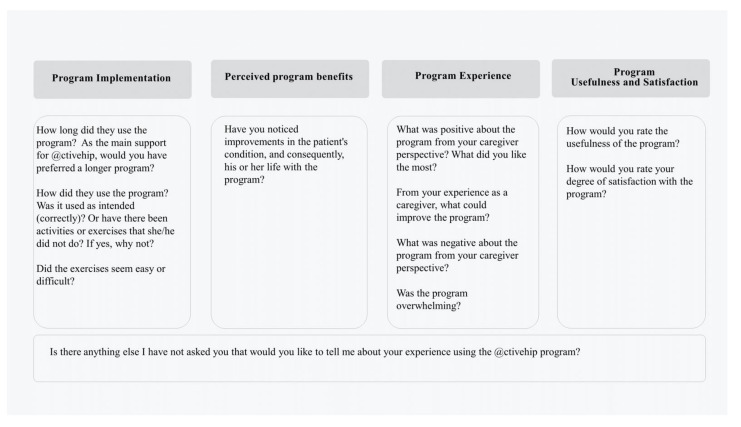
Interview guide.

## Data Availability

Not applicable.
